# Puerarin alleviates oxaliplatin-induced neuropathic pain by promoting Nrf2/GPX4-mediated antioxidative response

**DOI:** 10.1371/journal.pone.0308872

**Published:** 2024-08-14

**Authors:** Changqi Dai, Fangshou Zhen, Liangzhu Yu, Shen Xin

**Affiliations:** 1 School of Pharmacy, Xianning Medical College, Hubei University of Science and Technology, Xianning, Hubei, China; 2 Department of Pharmacy, Matang Hospital of Traditional Chinese Medicine, Xianning, Hubei, China; 3 School of Stomatology and Ophthalmology, Xianning Medical College, Hubei University of Science and Technology, Xianning, Hubei, China; The Second Affiliated Hospital of Guangzhou Medical University, CHINA

## Abstract

Oxaliplatin (OXA) as the platinum-based agent induces the cumulative neuropathy including functional impairment and neuropathic pain. OXA treatment triggered oxidative stress and inflammatory reaction in the spinal cord. Puerarin as a natural product has the neuroprotective effect on neuropathic pain. Hence, the roles and mechanisms of Pue on OXA induced neuropathic pain were studied. In this study, OXA-induced neuropathic pain mouse model was constructed by oxaliplatin injection for 5 consecutive days and two cycles. Pue (10 mg/kg) was administered intraperitoneally for seven consecutive days. The changes of behavior, morphology and levels of related proteins were detected. As a result, OXA-induced mice exhibited as the increased pain hypersensitivity, the impaired motor coordination, the activated NLRP3 inflammasome mediated inflammation and the suppressed nuclear factor erythroid 2-related factor 2 (Nrf2) mediated antioxidative reaction in the spinal cord (*P*<0.05 vs Control). After Pue administration, the mechanical pain threshold, thermal pain latency, spontaneous pain number and motor latency were improved (*P*<0.05 vs OXA). In the spinal cord, Pue administration reduced the levels of inflammatory elements, increased the levels of antioxidative elements and decreased the levels of oxidative factors (*P*<0.05 vs OXA). Furthermore, Pue also bind with Nrf2 and increased the association of Nrf2 to glutathione peroxidase 4 (GPX4). In summary, Pue alleviates oxaliplatin induced neuropathic pain by enhancing Nrf2/GPX4-mediated antioxidant response and suppressing inflammatory reaction in the spinal cord.

## Introduction

Oxaliplatin (OXA) is a widely used platinum-based agent for the chemotherapeutic eradication of cancer [1]. However, it has been reported to induce the acute or chronic neuropathy [2]. Aporoximately 85% of patients who are receiving oxaliplatin treatment experience the acute neuropathy, while approximately 15% of patient experiments cumulative chronic neuropathy [3]. Acute neuropathy is usually resolved within a few days, but cumulative neuropathy lasts for several years beyond the chemotherapy treatment causing functional impairment and neuropathic pain that has a negatively affect on the life quality of cancer survivors [4]. The American Society of Clinical Oncology recommends duloxetine as the agent to manage OXA-induced neuropathy [3]. Understanding the mechanisms underlying OXA-induced neuropathy is useful for developing a therapeutic strategy for OXA-induced neruopathic pain management.

OXA inhibits DNA replication and tumor cell proliferation by binding with DNA and forming DNA-platinum adducts. However, OXA treatment results in peripheral neurological damage by up-taking into dorsal root ganglia neurons and causes the deposition of platinum in spinal cord [5] which increases oxidative stress, glial activation and inflammatory reaction [6]. Oxidative stress is an imbalance between reactive oxygen species (ROS) production and antioxidants, and leads to chronic inflammation [7]. Antioxidants treatment reduces redox alteration and prevents neuropathic pain [8, 9]. In addition, the inhibition of astrocytic associated neuroinflammatory response by baicalein also improves the OXA-induced neuropathic pain [10]. Therefore, targeting the antioxidative response in the spinal cord may be a therapeutic method for OXA-induced neuropathic pain.

Puerarin (Pue) as a hydroxyisoflavone is a natural product isolated from *Neustanthus phaseoloides* (Roxb.) Benth, *Clematis hexapetala Pall*, and other organisms [11]. It possesses a broad spectrum of pharmacological properties including anti-diabetics, anti-hypertensive, anti-inflammation, anti-oxidant, and anti-tumor activities [12]. Pue has an alleviating effect on neuropathic pain in chronic constriction injury [13], diabetic neuropathy and paclitaxel-induced neuropathic pain models [14]. Pue has been reported to have a protecting effect on oxidative damage in the liver [15] and cerebral ischemia-reperfusion injury [16] through activating the antioxidant nuclear factor erythroid 2-related factor 2 (Nrf2) signaling pathway. Pue has an effect on obesrity-induced inflammation and dyslipidemia by decreasing the macrophages population and tumor necrosis factor-α (TNF-α) expression [17]. Puerarin also alleviates oxidative stress and inflammation by targeting NOD-, LRR-and pyrin domain-containing protein 3 (NLRP3) signaling [18]. However, the mechanism of pue on OXA-induced neuropathic pain remains unclear. Hence, in the present study, an OXA-induced neuropathic pain mouse model was constructed. Pue (10 mg/kg) was administered intraperitoneally for seven consecutive days. The changes of spinal inflammation, oxidative stress and pain behaviors were detected. This study aims to reveal the pathogenesis of OXA-induced neuropathic pain, and provide the supporting for the application of Pue in neuropathic pain.

## Materials and methods

### Chemicals and regents

Pue (B20446) was purchased from Shanghai yuanye (Shanghai, China). OXA (S1224) was purchased from Sellck (Shanghai, China). Anti-GFAP (A0237), anti-IL-1β (A19635), anti-NLRP3 (DF7438), anti-Caspase-1 (AF4005), anti-NRF2 (AF0639), anti-DHODH (DF3391), anti-GPX4 (DF6701), anti-β-actin (AF7018), HRP goat anti-rabbit IgG (H+L) (AS014) were purchased from Affinity (Jiangsu, China). Goat anti-mouse IgG H&L (FITC) (ab6785) and goat anti-rabbit IgG H&L (FITC) (ab6717) were purchased from Abcam (Cambridge, UK). The Reactive Oxygen Species Assay Kit (S0103), RIPA lysis buffer (BL504A), QuickBlock^™^ Blocking Buffer (P0260), CuZn/Mn SOD assay kit with WST 8 (S0103) and MDA kti (S0131S) were obtained from Beyotime (Shanghai, China). H&E staining solution (BL700A) and antigen repair solution (BL619A) was purchased from Biosharp (Shanghai, China).

### Animals and groups

Male C57BL/6J mice (6–8 weeks old, 18–20 g) were performed from Hubei Province Experimental Animal Centre (Wuhan, China), kept under 12 h light/dark environment and unlimited access to food and water. The experiment was approved by the Laboratory Animal Ethics Committee of Hubei University of Science and Technology (2021-05-981). Mice were randomly divided into Control, OXA, and OXA + Pue groups, ten mice for each group.

### Model construction and drug administration

OXA model group animals were intraperitoneal (i.p.) injected with 1 ml OXA (3 mg/kg, dissolved in 5% glucose solution, Selleck, Houston, USA) for 5 consecutive days, followed by 2 days of rest, for two cycles [19, 20]. Control group animals were injected with the same volume of 5% glucose solution. On the 15-21^th^ day after OXA injection, mice from OXA, and OXA + Pue were intraperitoneally injected with vehicle and puerarin (10 mg/kg), respectively, for seven consecutive days [21]. Pue was dissolved in DMSO and diluted with 0.9% NaCl before used. Vehicle was a mixture of DMSO and 0.9% NaCl (1:9). Behaviors tests were performed on day 0, 7, 14 and 21.

### Behavioral tests

Mechanical pain threshold test was used to evaluate the mechanical pain sensitivity and performed as follow. Mice were housed in a plexiglass container for almost 30 min. Von Frey filaments (Stoelting, Wood Dale, USA) were applied for stimulating the left hind paw. The filaments were briefly bent by being pushed firmly vertically on the plantar surfaces for 3–5 seconds. Paw flinching and brisk withdrawal were regarded as positive responses in this circumstance. The patterns of withdrawal responses were then translated into paw withdrawal threshold (PWT) values [22].

Spontaneous flinch test was used to evaluate the spontaneous pain and performed as follow. Mice were housed for nearly 30 minutes in a plexiglass chamber. Within 5 min, numbers of flinches were counted 3 times individually [23].

Paw withdrawal latency (PWL) test was used to evaluate the thermal pain hypersensitivity and performed as follow. After acclimatization, the light irradiation (temperature 45–65°C) stimulated the left hind paw until a withdrawal response occurs, with the cut-off time set to 20 sec to avoid tissue damage [24].

Rotarod test was used to evaluate the motor coordination and performed as follow. Mice were trained three days at a fixed pace for 10 minutes before the examinations. At experiments, the test was initially set at a constant speed at 10 revolutions per minute for 10 seconds, then at an increasing speed to 20 revolutions per minute for 30 seconds. The latency to fall was recorded [25].

### H&E staining

Following the behavior test, mice were given a deep anesthetic dose of 60 mg/kg sodium pentobarbital and transcardially perfused using 4% PFA. After collected and post-fixed for 12 hours at 4°C, the spinal cords were embedded with paraffin and cutting into 4-μm sections. The sections were treated with xylene, ethanol and dyed with H&E staining kit. Then the sections were sealed and observed with the fluorescence microscope (IX73, Olympus, Tokyo, Japan) and analyzed using ImageJ 1.53a. Inflammatory cell infiltration was categorized as 0 (normal); 1 (meningeal and perivascular lymphocytic infiltration); 2 (1–10 lymphocytes present); 3 (11–100 lymphocytes); 4 (>100 lymphocytes).

### Immunofluorescence assay

The sections of spinal cord tissue were hydrated and subjected to antigen retrieval using antigen retrieval solution for 10 min at 95°C. The sections were blocked with immunofluorescence blocking solution for 1 hour before incubating with primary antibodies (1:100) at 4°C overnight and fluorescent secondary antibodies for 1 hour at room temperature. The images were captured by a fluorescence microscope and analyzed using ImageJ 1.53a.

### Western blotting assay

Mice were decapitated and intraperitoneally injected with 150 mg/kg pentobarbital sodium after the behavioral tests. The lumbar spinal cords were homogenized in RIPA lysis buffer and centrifugated for 20 min (13,523 x g, 4°C), The supernatant was isolated with SDS-PAGE and transferred on PVDF membranes. The blots were blocked using blocking buffer, incubated with primary antibodies (1:1000) overnight at 4°C and secondary antibody (1:5,000) for one hour at room temperature, visualized using ECL solution, detected with an iBright 1500 instrument (Invitrogen, CA, USA) and analyzed by ImageJ 1.53a.

### Manganese superoxide dismutase (Mn-SOD) activity detection

The Mn-SOD assay kit was employed to measure the Mn-SOD activity. Shortly, after homogenizing in PBS and centrifuging at 13,523 x g for 15 min, the supernatant of spinal cord was collected and incubated with Cu/Zn Mn-SOD inhibitors for one hour at 37°C following with WST-8 enzyme working solution for 20 min at 37°C, and measured the OD_450 nm_ absorbance using Synergy HTX Microplate Reader (BioTek, VT, USA).

### Malondialdehyde (MDA) measurement

After homogenizing and centrifuging, the supernatants of spinal cord were treated using MDA kti by incubating with working solution and measuring the OD_532 nm_ absorbance using Synergy HTX Microplate Reader (BioTek, VT, USA).

### Molecular docking

Nrf2 X-ray crystal structure (PDB ID: 4L7D) was acquired through Protein Data Bank. The structure of puerarin was optimized and retrieved from the PubChem compound database (PubChem CID: 5281807). The docking conformation of puerarin and Nrf2 was determined using Auto Dock Vina 1.2.0, and visualized with PyMOL 2.2.3 (20).

### Statistical analysis

All statistical analyses were employed using SPSS 26.0 statistics software. Two-way ANOVA analysis was used to assess the differences between groups in the behavioral tests. And one-way ANOVA analysis was used to assess the differences between groups in tests of H&E, immunofluorescence, Western blotting, Mn-SOD activity and MDA measurement. Statistical significance was set at P < 0.05.

## Results

### Pue decreases pain sensitivity in OXA-induced mice

**Fig 1:**
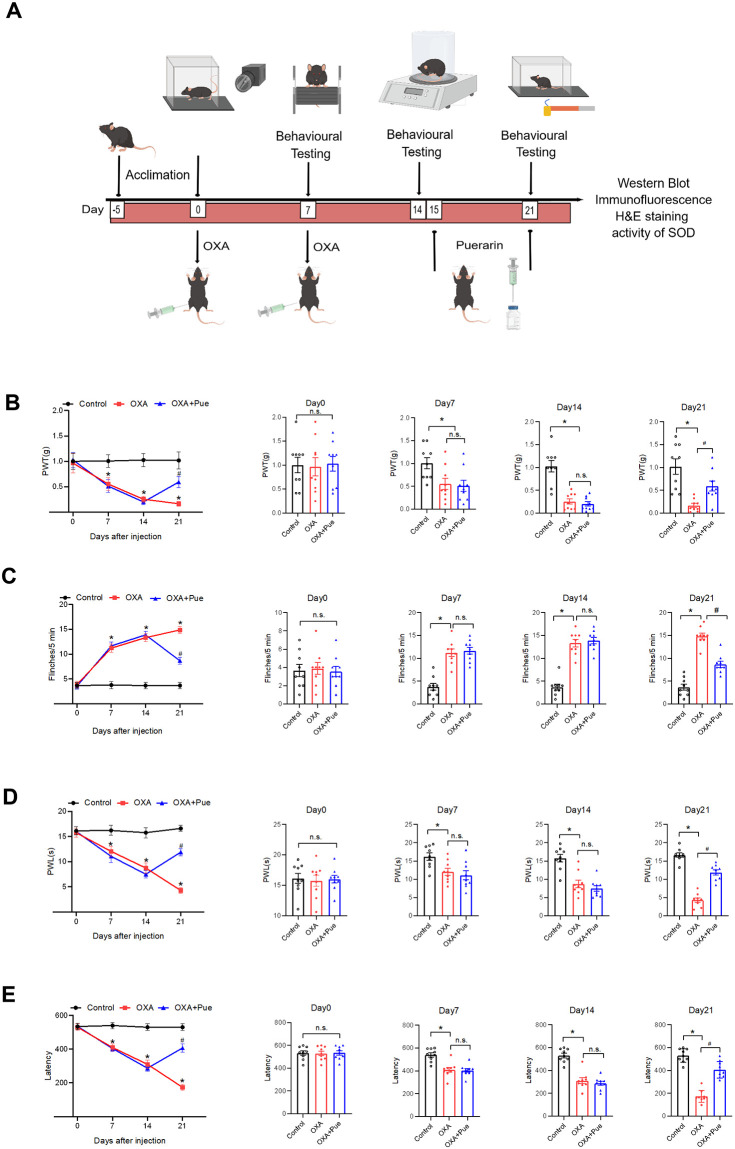
Effect of Pue on pain behaviors. (A) Schematic diagram of the experimental procedures. (B-E) Changes of PWT values, flinches number, PWL time and latency to fall in mice. Data are presented as the mean ± SEM (n = 9). *P < 0.05 vs. control group, ^#^P < 0.05 vs. OXA group. PWT, paw withdrawal threshold; PWL, paw withdrawal latency; OXA, oxaliplatin, Pue, puerarin.

Behavioral tests were performed following the protocol as shown in Fig 1A. On day 14, compared with control group, OXA inducement statistically increased mechanical pain presenting as the decreased PWT values from 1.03 ± 0.13 to 0.26 ± 0.06 (P < 0.05, Fig 1B), increased spontaneous pain presenting as the elevated flinch numbers from 3.67 ± 0.65 to 13.33 ± 0.83 (P < 0.05, Fig 1C), increased thermal hyperalgesia presenting as the reduced PWL values from 15.77 ± 1.03 to 8.73 ± 1.04 (P < 0.05, Fig 1D), and decreased motor coordination presenting as the lowered latency to fall from 530.31 ± 19.81 to 310.38 ± 24.87 (P < 0.05, Fig 1E). After Pue administration, OXA + Pue group mice showed a raised PWT value at 0.59 ± 0.11 (P < 0.05 vs OXA, Fig 1B), a decreased flinches numbers of 8.67 ± 0.67 (P < 0.05 vs OXA, Fig 1C), an increased PWL values at 11.89 ± 0.69 (P < 0.05 vs OXA, Fig 1D), and a recovered latency to fall of 407.15 ± 24.51 (P < 0.05 vs OXA, Fig 1E). These data illustrated that Pue administration reduced mechanical pain, spontaneous pain and thermal hyperalgesia, and recovered motor coordination on OXA-induced mice.

### Pue suppresses inflammation in the spinal cord of OXA-induced mice

**Fig 2:**
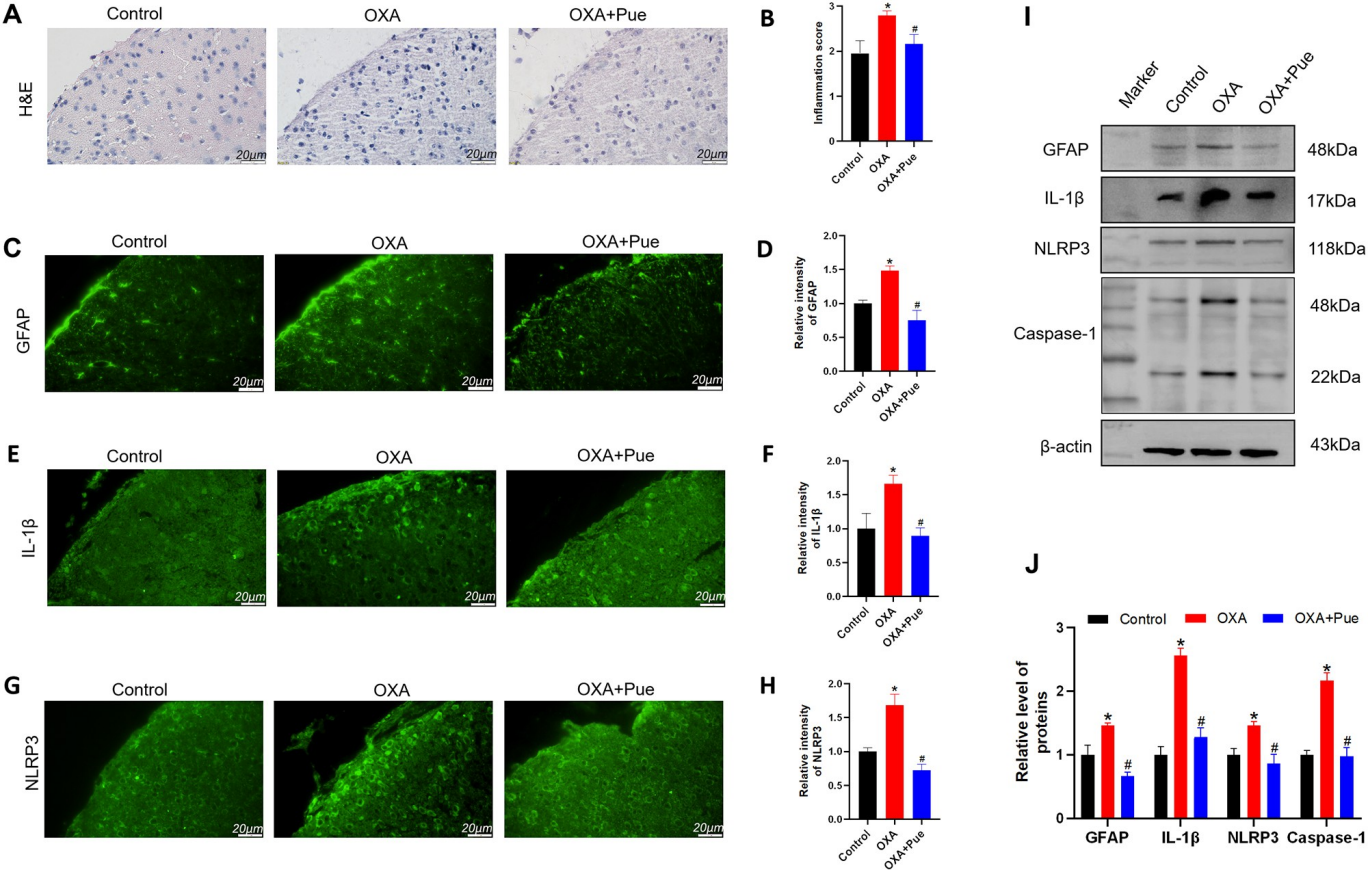
Effect of Pue on the spinal inflammation. (A and B) Representative images of H&E staining (A) and quantitative analysis (B) of inflammation in the spinal cord. (C and D) Representative images of immunofluorescence staining and quantitative analysis of GFAP in the spinal cord. (E and F) Representative images of immunofluorescence staining and quantitative analysis of IL-1β in spinal cord sections. (G and H) Representative images of immunofluorescence staining and quantitative analysis of NLRP3 in the spinal cord. Scale bar = 20 μm. (I and J) Representative bands of western blotting and quantitative analysis of GFAP, IL-1β, NLRP3 and Caspase-1 expression levels in the spinal cord. Data are presented as the mean ± SD (n = 3). *P < 0.05 vs. control group, ^#^P < 0.05 vs. OXA group. H&E, hematoxylin and eosin; GFAP, glial fibrillary acidic protein; NLRP3, NOD-like receptor protein 3 inflammasome; IL-1β, interleukin-1β; OXA, oxaliplatin; Pue, puerarin.

Inflammatory cell infiltration was increased in the spinal dorsal horn of OXA group detecting by H&E staining (Fig 2A). The inflammatory score in OXA group was increased to 2.79 ± 0.11, while control group at 1.95 ± 0.28 (P < 0.05, Fig 2B). Pro-inflammatory cytokine interleukin-1β (IL-1β) level in the spinal cord of OXA group was elevated with a relative intensity of 1.66 ± 0.12 (P < 0.05 vs control, Fig 2E and 2F) and the relative gray value of 2.56 ± 0.11 (P < 0.05 vs control, Fig 2I and 2J). Astrocytes are known source of IL-1β releasing in persistent pain. The intensity and expression level of glial fibrillary acidic protein (GFAP) as a marker of astrocyte in the spinal dorsal horn of OXA group was significantly increased with a relative intensity value of 1.67 ± 0.12 (P < 0.05 vs control, Fig 2C and 2D) and a relative gray value of 1.46 ± 0.04 (P < 0.05 vs control, Fig 2I and 2J). NLRP3 inflammasome activation is essential for caspase-1 cleavage and IL-1β maturation. The NLRP3 level in spinal cord of OXA group was elevated with a relative intensity of 1.69 ± 0.16 (P < 0.05 vs control group, Fig 2G and 2H) and a relative gray value of 1.46 ± 0.06 (P < 0.05 vs control, Fig 2I and 2J). While, the expression of caspase-1 was increased in OXA group with the gray value at 2.17 ± 0.12 (P < 0.05 vs control, Fig 2I and 2J). After Pue administration, the inflammatory cells infiltration in spinal cord was decreased (P < 0.05 vs OXA), and the intensities and expression levels of IL-1β, GFAP, NLRP3 and caspase-1 in spinal cord were reduced (P < 0.05 vs OXA).

### Pue stimulates anti-oxidative response in the spinal cord of OXA-induced mice

**Fig 3:**
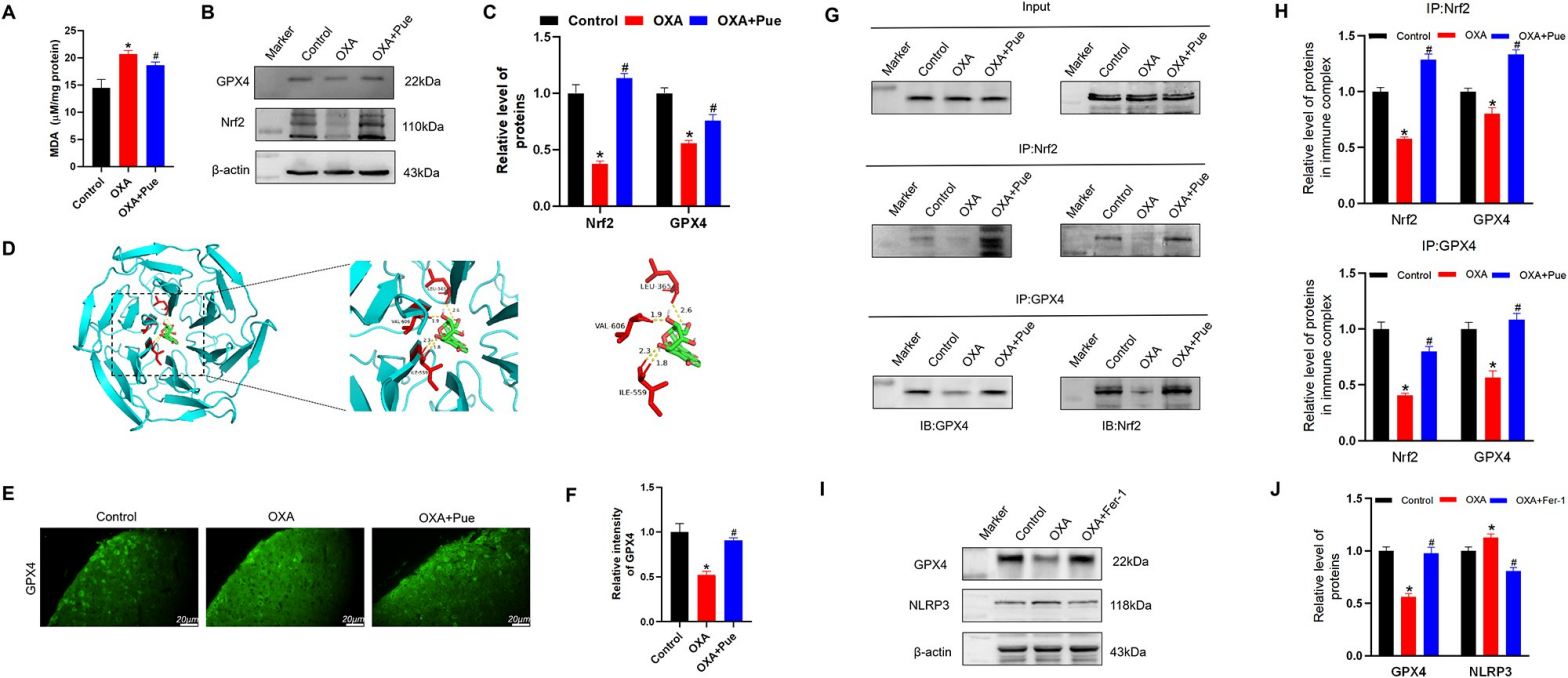
Effect of Pue on the oxidative damage. (A) The MDA level in spinal cord of mice. Scale bar = 20 μm. (B and C) Representative bands of western blotting and quantitative analysis for GPX4 and Nrf2 expression levels in the spinal cord. (D) The molecular docking images of Nrf2 with Pue. (E and F) Representative images of immunofluorescence staining and quantitative analysis for GPX4 in the spinal cord. (G and H) Representative bands of immunoprecipitation and quantitative analysis for the interaction between Nrf2 and GPX4. (I and J) Representative bands of western blotting and quantitative analysis for GPX4 and NLRP3 expression levels in the spinal cord after Fer-1 treatment. Data are presented as the mean ± SD (n = 3). *P < 0.05 vs. control group, ^#^P < 0.05 vs. OXA group. GPX4, glutathione peroxidase 4; Nrf2, nuclear factor erythroid 2-related factor 2; NLRP3, NOD-like receptor protein 3 inflammasome; MDA, malondialdehyde; Fer-1, ferrostatin-1; OXA, oxaliplatin; Pue, puerarin.

Oxidative reaction directly and indirectly promotes inflammation activation [26]. As the biological markers of oxidative stress [27], MDA level in the spinal cord of control group was at 14.51 ± 1.54 μmol/mg. While, it increased to 22.75 ± 0.59 μmol/mg in OXA group (P < 0.05 vs control, Fig 3A). Nrf2 is a key antioxidant response element, and its expression level in the spinal cord of the OXA group was decreased, with a relative gray value of 0.38 ± 0.03 (P < 0.05 vs control, Fig 3B and 3C). Autodock data showed that Pue formed four electrovalent bonds with Nrf2 at VAL-606, ILE559, and LEU-365 with the binding affinity at -9.7 kcal/mol (Fig 3D). Glutathione peroxidase 4 (GPX4) is a main antioxidant mediator and its synthesis and functions were directly affected by Nrf2 [28]. The intensity and expression level of GPX4 in the spinal cord of OXA group was significantly decreased with a relative intensity value of 0.52 ± 0.04 (P < 0.05 vs control, Fig 3E and 3F) and a relative gray value of 0.56 ± 0.02 (P < 0.05 vs control, Fig 3B and 3C). After Pue administration, the MDA level reduced to 18.67 ± 0.58 (P < 0.05 vs OXA, Fig 3A), the intensity and expression levels of Nrf2 and GPX4 were increased (P < 0.05 vs OXA, Fig 3B and 3C). Immunoprecipitation was performed to confirm the effect of Pue on the interaction between Nrf2 and GPX4. As shown in Fig 3G, input represented a positive control to demonstrate the existence of Nrf2 and GPX4 in the spinal cord tissue extracts. The immunoprecipitants bands presented that the interaction between Nrf2 and GPX4 was decreased in the spinal cords of the OXA group (P<0.05 vs. control). Pue treatment increased the association of Nrf2 to GPX4 (P<0.05 vs. OXA). Further, Fer-1 was used to restore the expression of GPX4, and as a result GPX4 level was increased, while the NLRP3 level was reduced in OXA group after Fer-1 treatment (P<0.05 vs. OXA, Fig 3I and 3G).

### Pue reduced mitochondrial-mediated ROS level in the spinal cord of OXA-induced mice

**Fig 4:**
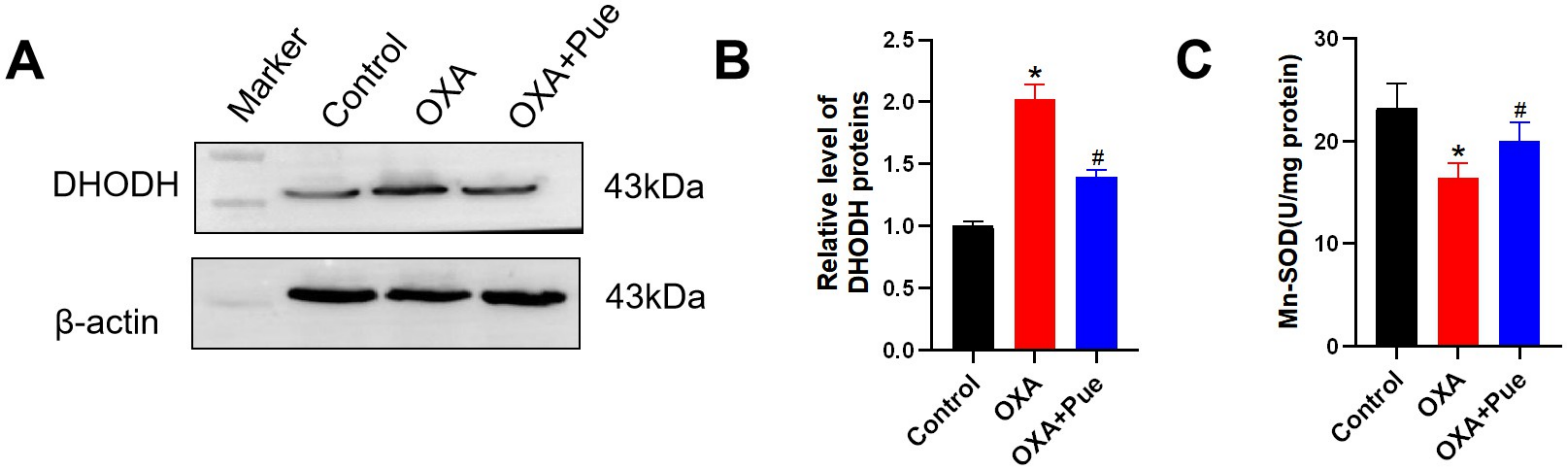
Effect of Pue on mitochondrial ROS level. (A and B) Representative bands of Western blotting and quantitative analysis for DHODH expression level in the spinal cord. (C) The activity of Mn-SOD in the spinal cord. Data are presented as the mean ± SD (n = 3). *P < 0.05 vs. control group, ^#^P < 0.05 vs. OXA group. DHODH, dihydroorotate dehydrogenase; ROS, reactive oxygen species; Mn-SOD, manganese superoxide dismutase; OXA, oxaliplatin; Pue, puerarin.

The mitochondria are the major source of reactive oxygen species (ROS) and dihydroorotate dehydrogenase (DHODH) is a major enzyme contributing to mitochondrial oxygen consumption and ROS production [29]. Here, the DHODH expression level in the spinal cord was significantly increased in OXA group with a relative gray value of 2.03 ± 0.11 (P < 0.05 vs control, Fig 4A and 4B). Mn-SOD is a principal antioxidant enzyme locating in mitochondria and governing the types of ROS [30]. Mn-SOD activity in the control group was at 23.28 ± 2.36 U/mg, however, it was suppressed to 16.46 ± 1.41 U/mg in the OXA group (P < 0.05 vs control, Fig 4C). After Pue administration, the intensity and level of mitochondrial DHODH were both decreased (P < 0.05 vs OXA, Fig 4A and 4B). While the Mn-SOD activity was increased to 20.00 ± 1.82 (P < 0.05 vs OXA, Fig 4C).

## Discussion

This study found that Pue treatment relieved OXA-induced neuropathic pain and activated Nrf2 mediated antioxidant response. Nrf2 activation exerts the analgesic effects in animal models of paclitaxel-induced neuropathic pain, chronic inflammatory and neuropathic pain [31, 32]. Nrf2 activation also plays a synergic function for opioids and morphine by enhancing the analgesic effects and reducing their side effects [33]. Nrf2 commonly works as the regulator for the antioxidant defense through binding with the antioxidant response elements and initiating the transcription of targeted genes [34]. Meanwhile, GPX4 as a central defense enzyme is a substrate of Nrf2 [35]. Nrf2 reduces the level of lipid peroxida and alleviates oxidative damage through regulating GPX4 expression [28]. Furthermore, mitochondria is the main source of peroxide production and mediates the reduction of oxygen single electrons to superoxide causing by the electron leakage from the electron respiratory chain complexes [36]. DHODH is found in the inner membrane of mitochondria, catalyzes the conversion of dihydroorotate to orotate and directly generates superoxida and H_2_O_2_ directly [37]. DHODH and GPX4 are essential metabolic enzymes for eliminating toxic lipid peroxides in the mitochondria [38]. And we found that in OXA-induced mice, the ROS levels in mitochondria and cytoplasm were increased. Pue is reported stabilizing mitochondrial membrane potential, reducing mitochondrial dysfunction and cellular ROS accumulation [39]. Pue also works on increasing the levels of antioxidative elements such as Nrf2 SOD and GPX4 [40]. In our study, we found Pue changed the levels of Nrf2, GPX4, DHODH, MDA and Mn-SOD. Thus, we suggested that Pue could reduce the oxidative damage and have the antinociceptive effect on neuropathic pain.

This study also found that puPue suppressesd NLRP3 mediated inflammation in the spinal cord of OXA-induced mice. Nrf2 is reported acts against NLRP3 inflammasome activation by regulating the thioredoxin1/thioredoxin interacting protein complex [41]. Nrf2 activation directly blocks the transcription and expression of the proinflammatory cytokines (TNF-α, IL-1β and IL-6) and the activation of NF-κB [42]. Pue is reported to up-regulate Nrf2 activity, reduce the levels of NLRP3 inflammasome complex and downstream IL-18 and IL-1β production [43]. Therefore, we suggested that Pue could play anti-inflammatory effect by Nrf2-NLRP3 pathway.

Although this study provides the effects of Pue on OXA-induced neuropathy pain, there are still some limitations. Firstly, Nrf2 is considered as the target protein for oxaliplatin induced neuropathic pain management in the present study, but the expression of Nrf2 on the genetic level in animals is not constructed and requires further study. Secondly, Nrf2 is suggested to be the target for Pue, and the binding between Pue and Nrf2 needs to be directly demonstrated in future experiments. Thirdly, Pue also has functions on OXA resistance and tumor suppression [44], this effect is required in future research.

## Conclusion

**Fig 5:**
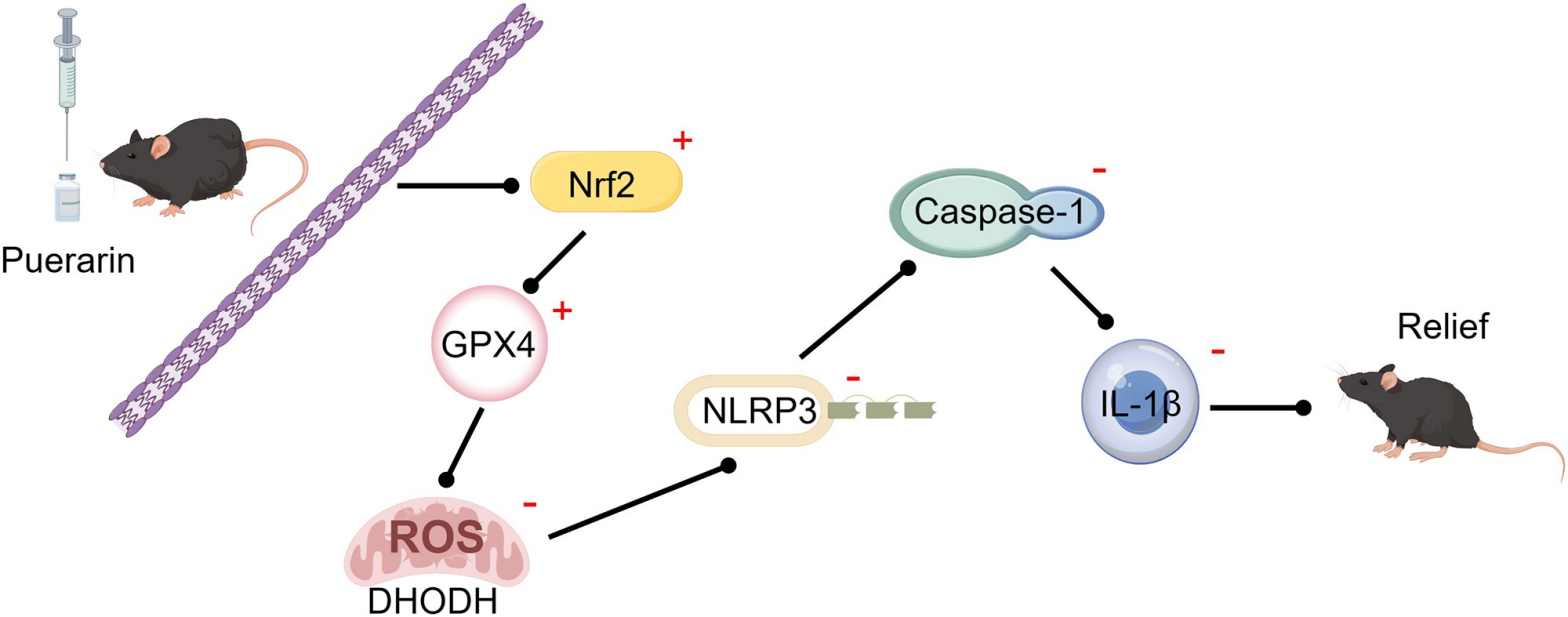
Schematic representation of the potential mechanisms of Pue on OXA-induced neuropathic pain. OXA, oxaliplatin; Pue, puerarin.

Following OXA inducement, the spinal inflammation is stimulated, oxidative damage is increased, and OXA-induced neuropathic pain is triggered. Pue administration enhances Nrf2/GPX4-mediated antioxidant response, suppresses inflammatory reaction, and alleviates OXA-induced neuropathic pain (Fig 5).

## Supporting information

S1 Table Summary of the results of the two-way ANOVA analysis for behavioral tests. (DOCX)

S1 Raw image (PDF)
