# Supplementary material for: Puerarin alleviates oxaliplatin-induced neuropathic pain by promoting Nrf2/GPX4-mediated antioxidative response
Source: PLoS One. 2024 Aug 14;19(8):e0308872. doi: 10.1371/journal.pone.0308872 (PMC11324108; doi:10.1371/journal.pone.0308872)
Supplement: S1 Table — (DOCX) [file pone.0308872.s001.docx]

S-Table 1. Summary of the results of the two-way ANOVA analysis for behavioral tests.

| Behavioral tests | Source | Sum of squares | df | Mean square | F | Sig. |
| --- | --- | --- | --- | --- | --- | --- |
| PWT | Intercept | 52.138 | 1 | 52.138 | 313.1 | 0.000** |
|  | Time | 3.857 | 3 | 1.286 | 7.72 | 0.000** |
|  | Group | 5.679 | 2 | 2.84 | 17.053 | 0.000** |
|  | Residual | 16.985 | 102 | 0.167 |  |  |
|  | R Square | 0.360 | | | | |
| PWL | Intercept | 17344.724 | 1 | 17344.724 | 1526.072 | 0.000** |
|  | Time | 489.191 | 3 | 163.064 | 14.347 | 0.000** |
|  | Group | 702.231 | 2 | 351.116 | 30.893 | 0.000** |
|  | Residual | 1159.291 | 102 | 11.366 |  |  |
|  | R Square | 0.507 | | | | |
| Numbers of flinches | Intercept | 6896.009 | 1 | 6896.009 | 765.961 | 0.000** |
|  | Time | 693.213 | 3 | 231.071 | 25.666 | 0.000** |
|  | Group | 1031.463 | 2 | 515.731 | 57.284 | 0.000** |
|  | Residual | 918.315 | 102 | 9.003 |  |  |
|  | R Square | 0.653 | | | | |
| Latency to fall | Intercept | 20230257.87 | 1 | 20230257.87 | 2673.559 | 0.000** |
|  | Time | 474463.796 | 3 | 158154.599 | 20.901 | 0.000** |
|  | Group | 600322.273 | 2 | 300161.137 | 39.668 | 0.000** |
|  | Residual | 771812.374 | 102 | 7566.788 |  |  |
|  | R Square | 0.582 | | | | |

* p<0.05, ** p<0.01

S-Table 2. Summary of the results of the one-way ANOVA analysis for WB, IF, IP and H&E assays.

| Assays | Source of variation | Sum of squares | df | Mean square | F | Sig. |
| --- | --- | --- | --- | --- | --- | --- |
| H&E  (Fig 2A) | Between Groups | 3.406 | 2 | 1.703 | 37.785 | 0.000** |
|  | Within Groups | 1.082 | 24 | 0.045 |  |  |
|  | Total | 4.488 | 26 |  |  |  |
| GFAP  (Fig. 2C) | Between Groups | 576.347 | 2 | 288.173 | 44.502 | 0.000** |
|  | Within Groups | 38.853 | 6 | 6.475 |  |  |
|  | Total | 615.2 | 8 |  |  |  |
| IL-1β  (Fig. 2E) | Between Groups | 364.130 | 2 | 182.065 | 19.259 | 0.000** |
|  | Within Groups | 56.722 | 6 | 9.454 |  |  |
|  | Total | 420.852 | 8 |  |  |  |
| NLRP3  (Fig. 2G) | Between Groups | 697.472 | 2 | 348.736 | 58.646 | 0.000** |
|  | Within Groups | 35.679 | 6 | 5.946 |  |  |
|  | Total | 733.150 | 8 |  |  |  |
| GFAP  (Fig. 2I) | Between Groups | 951557089.7 | 2 | 475778544.9 | 50.403 | 0.000** |
|  | Within Groups | 56637238.00 | 6 | 9439539.666 |  |  |
|  | Total | 1008194328 | 8 |  |  |  |
| IL-1β  (Fig. 2I) | Between Groups | 1049789992 | 2 | 524894996.1 | 120.524 | 0.000** |
|  | Within Groups | 26130682.88 | 6 | 4355113.814 |  |  |
|  | Total | 1075920675 | 8 |  |  |  |
| NLRP3  (Fig. 2I) | Between Groups | 672645403.3 | 2 | 336322701.6 | 26.273 | 0.001** |
|  | Within Groups | 76807553.73 | 6 | 12801258.95 |  |  |
|  | Total | 749452957.0 | 8 |  |  |  |
| Caspase-1  (Fig. 2I) | Between Groups | 477350427.8 | 2 | 238675213.9 | 161.635 | 0.000** |
|  | Within Groups | 8859778.309 | 6 | 1476629.718 |  |  |
|  | Total | 486210206.1 | 8 |  |  |  |
| MDA  (Fig. 3A) | Between Groups | 61.185 | 2 | 30.592 | 28.819 | 0.001** |
|  | Within Groups | 6.369 | 6 | 1.062 |  |  |
|  | Total | 67.554 | 8 |  |  |  |
| GPX4  (Fig. 3B) | Between Groups | 549387469.6 | 2 | 274693734.8 | 77.071 | 0.000** |
|  | Within Groups | 21384885.60 | 6 | 3564147.600 |  |  |
|  | Total | 570772355.2 | 8 |  |  |  |
| Nrf2  (Fig. 3B) | Between Groups | 809999151.9 | 2 | 404999576.0 | 46.821 | 0.000** |
|  | Within Groups | 51899457.27 | 6 | 8649909.544 |  |  |
|  | Total | 861898609.2 | 8 |  |  |  |
| GPX4  (Fig. 3E) | Between Groups | 639.703 | 2 | 319.852 | 49.515 | 0.000** |
|  | Within Groups | 38.758 | 6 | 6.460 |  |  |
|  | Total | 678.461 | 8 |  |  |  |
| GPX4  (Fig. 3G) | Between Groups | 276527447.2 | 2 | 138263723 | 118.643 | 0.000** |
|  | Within Groups | 6992257.701 | 6 | 1165376.283 |  |  |
|  | Total | 283519704.9 | 8 |  |  |  |
| GPX4  (Fig. 3G) | Between Groups | 228496281.5 | 2 | 114248140.8 | 71.562 | 0.000** |
|  | Within Groups | 9578926.023 | 6 | 1596487.671 |  |  |
|  | Total | 238075207.5 | 8 |  |  |  |
| Nrf2  (Fig. 3G) | Between Groups | 749049087.8 | 2 | 374524543.9 | 286.807 | 0.000** |
|  | Within Groups | 7835057.413 | 6 | 1305842.902 |  |  |
|  | Total | 756884145.2 | 8 |  |  |  |
| Nrf2  (Fig. 3G) | Between Groups | 1000539211 | 2 | 500269605.6 | 136.601 | 0.000** |
|  | Within Groups | 21973604.00 | 6 | 3662267.333 |  |  |
|  | Total | 1022512815 | 8 |  |  |  |
| GPX4  (Fig. 3I) | Between Groups | 668351175.0 | 2 | 334175587.5 | 96.423 | 0.000** |
|  | Within Groups | 20794270.45 | 6 | 3465711.742 |  |  |
|  | Total | 689145445.4 | 8 |  |  |  |
| NLRP3  (Fig. 3I) | Between Groups | 229482860.5 | 2 | 114741430.3 | 59.592 | 0.000** |
|  | Within Groups | 11552661.63 | 6 | 1925443.606 |  |  |
|  | Total | 241035522.1 | 8 |  |  |  |
| DHODH  (Fig. 4A) | Between Groups | 1215782135 | 2 | 607891067.5 | 132.806 | 0.000** |
|  | Within Groups | 27463659.09 | 6 | 4577276.515 |  |  |
|  | Total | 1243245794 | 8 |  |  |  |
| Mn-SOD  ((Fig. 4C) | Between Groups | 116.559 | 2 | 58.279 | 16.056 | 0.000** |
|  | Within Groups | 43.558 | 12 | 3.630 |  |  |
|  | Total | 160.117 | 14 |  |  |  |

*p<0.05, **p<0.01.
