# Supplementary figures and images for: Puerarin alleviates oxaliplatin-induced neuropathic pain by promoting Nrf2/GPX4-mediated antioxidative response
Source: PLoS One. 2024 Aug 14;19(8):e0308872. doi: 10.1371/journal.pone.0308872 (PMC11324108; doi:10.1371/journal.pone.0308872)

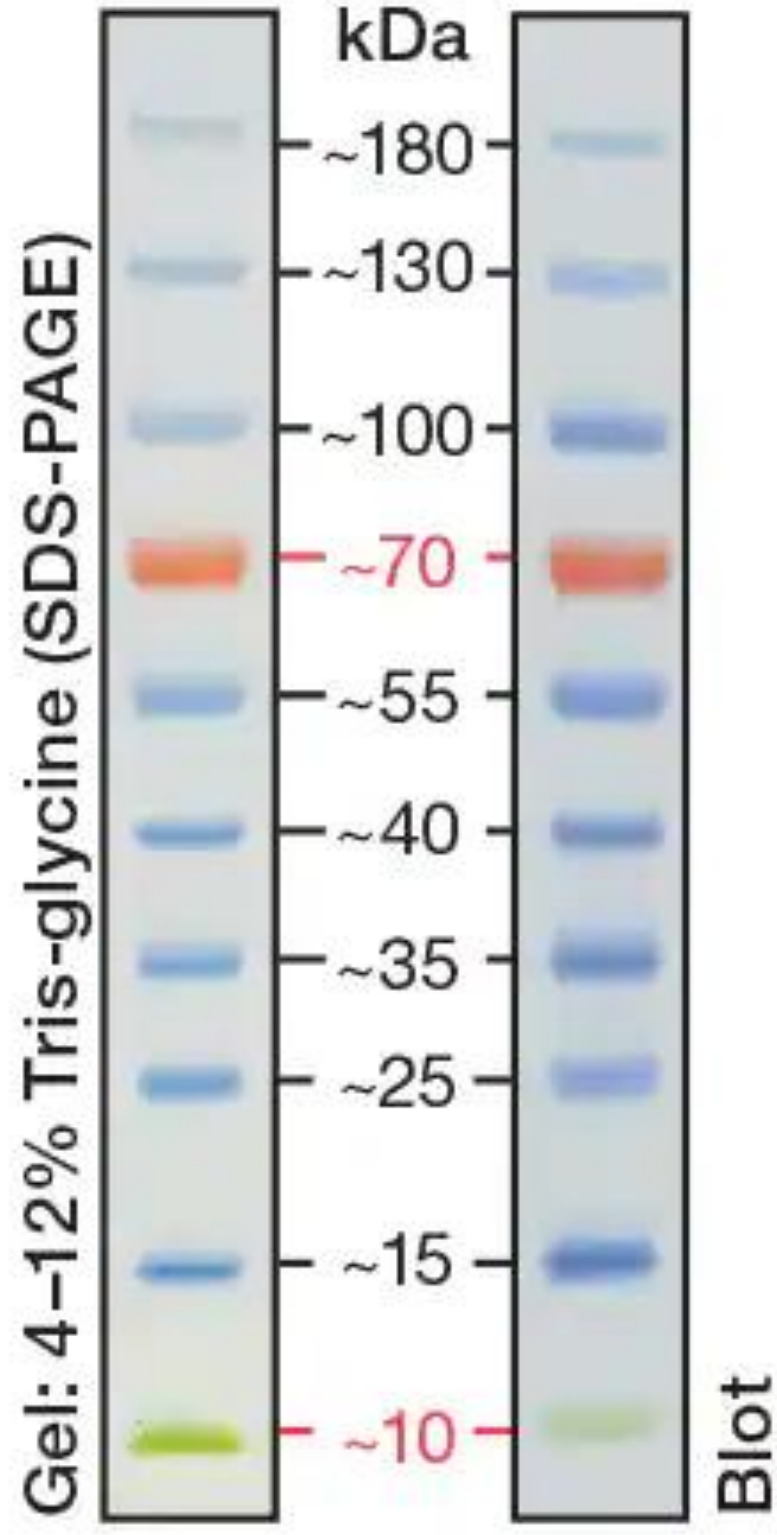

Fig2

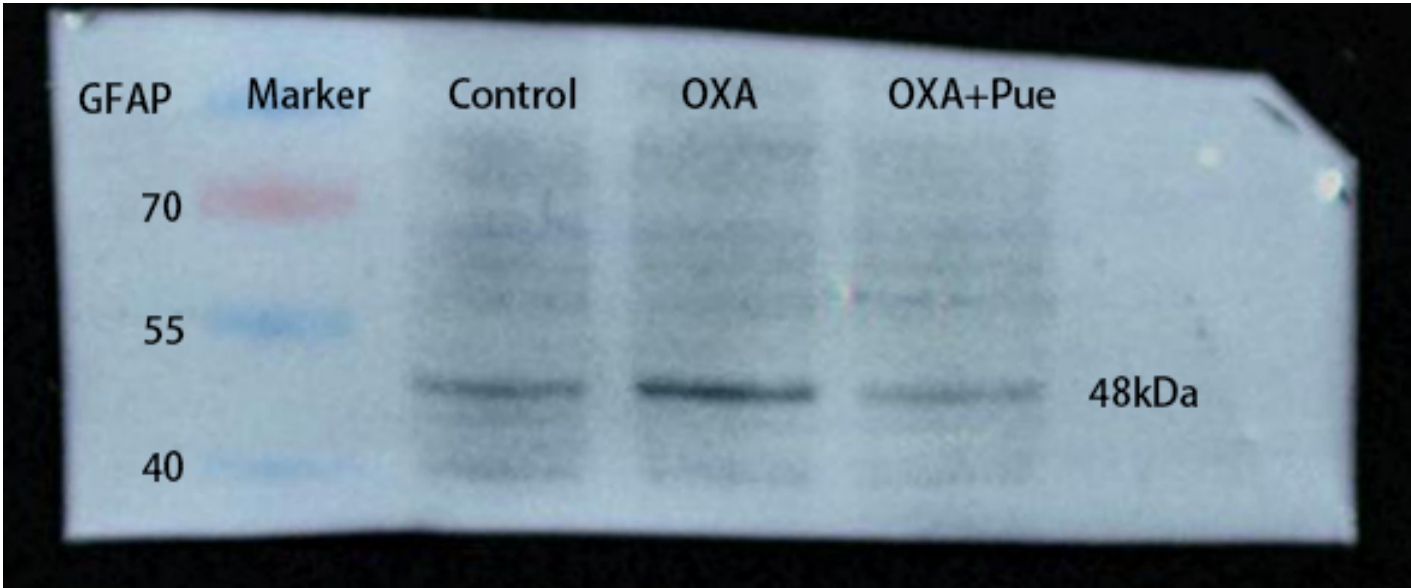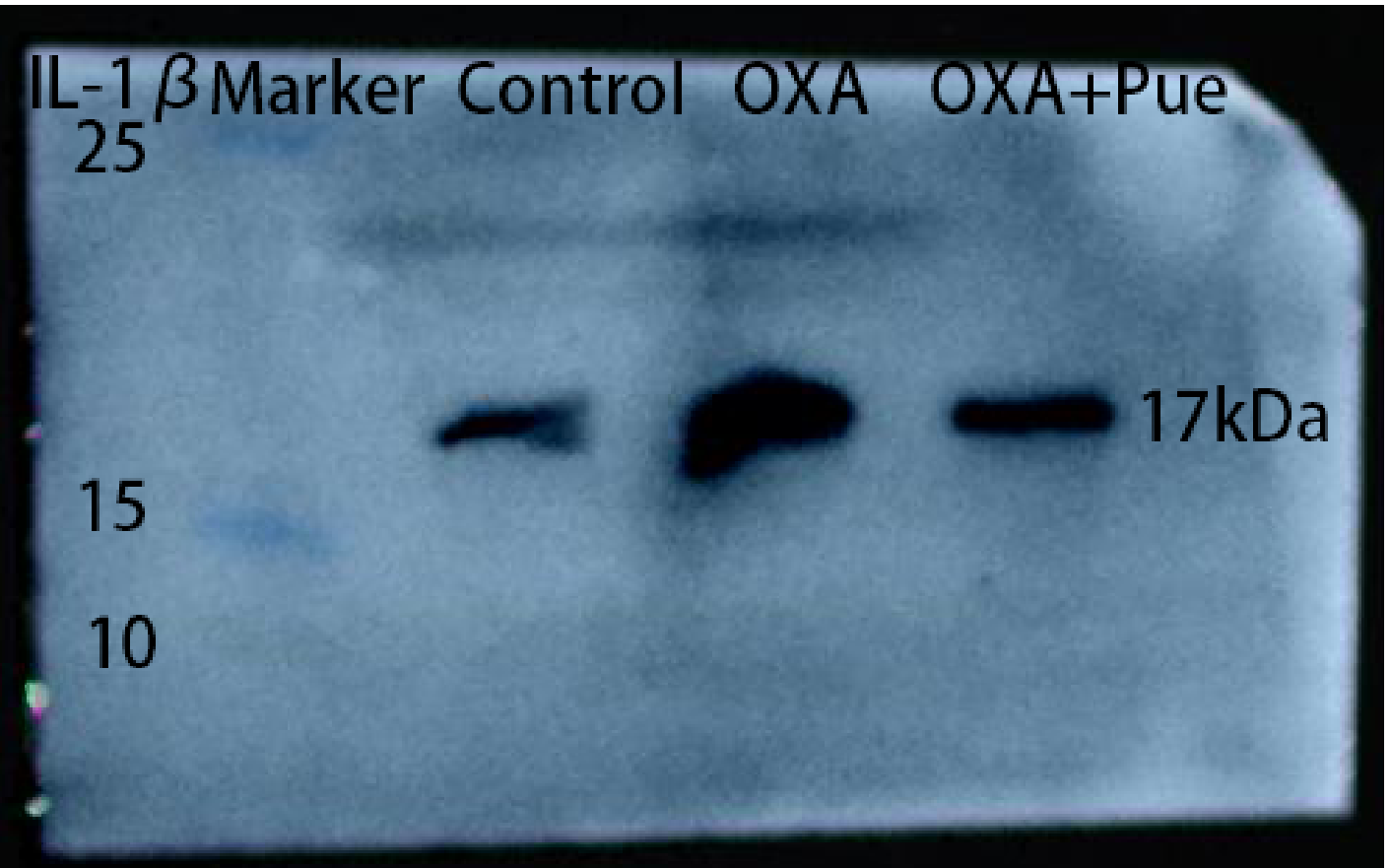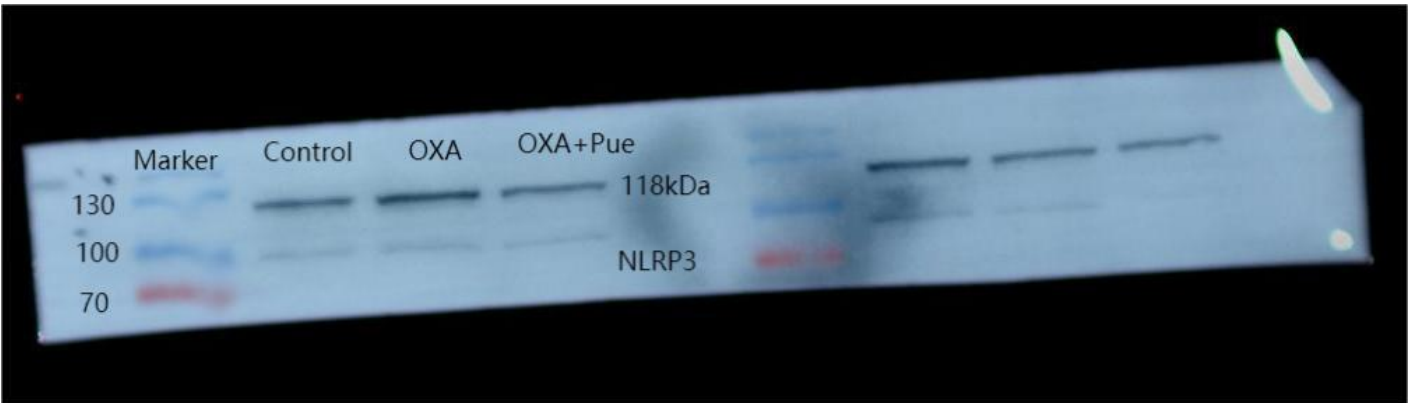

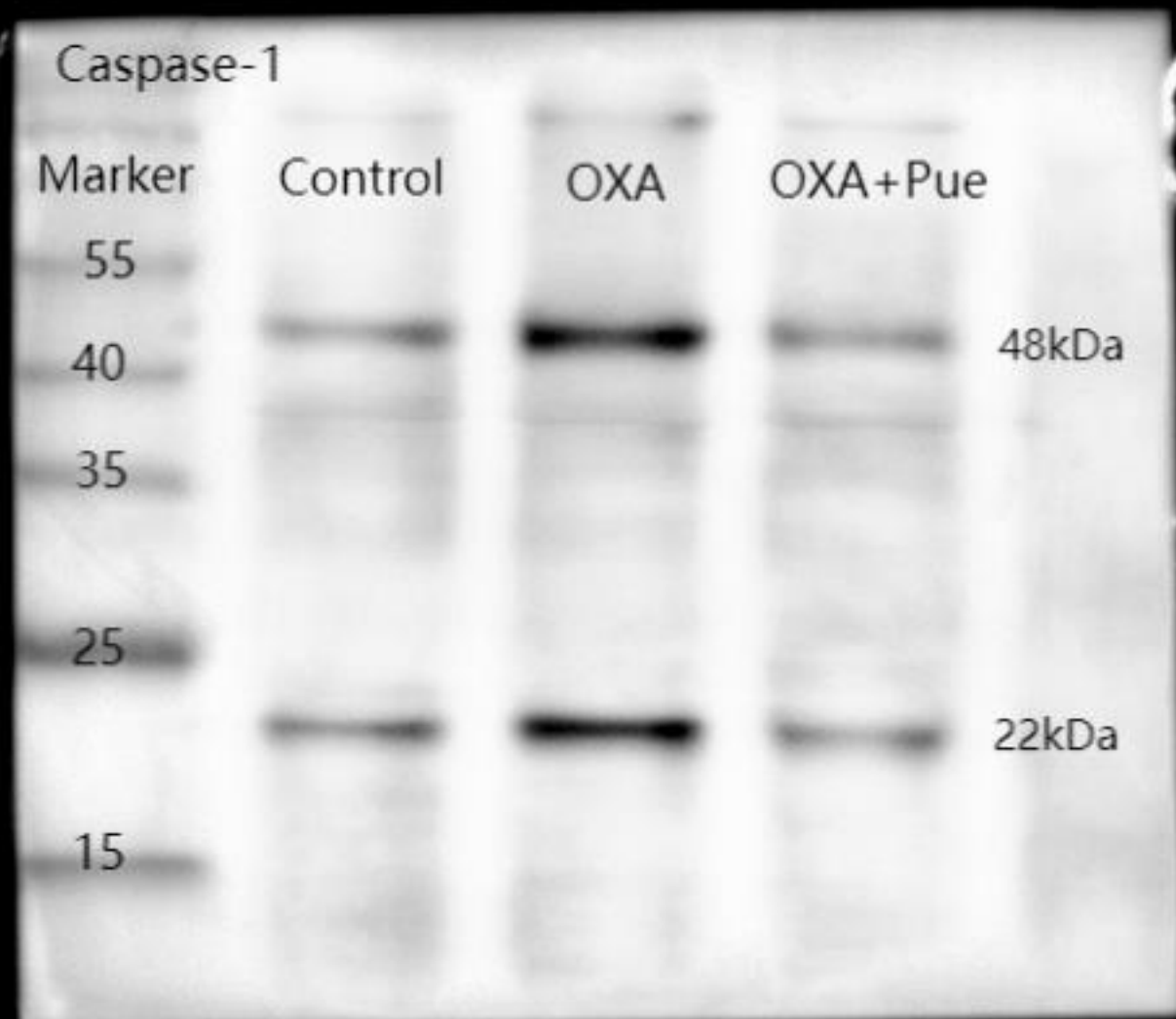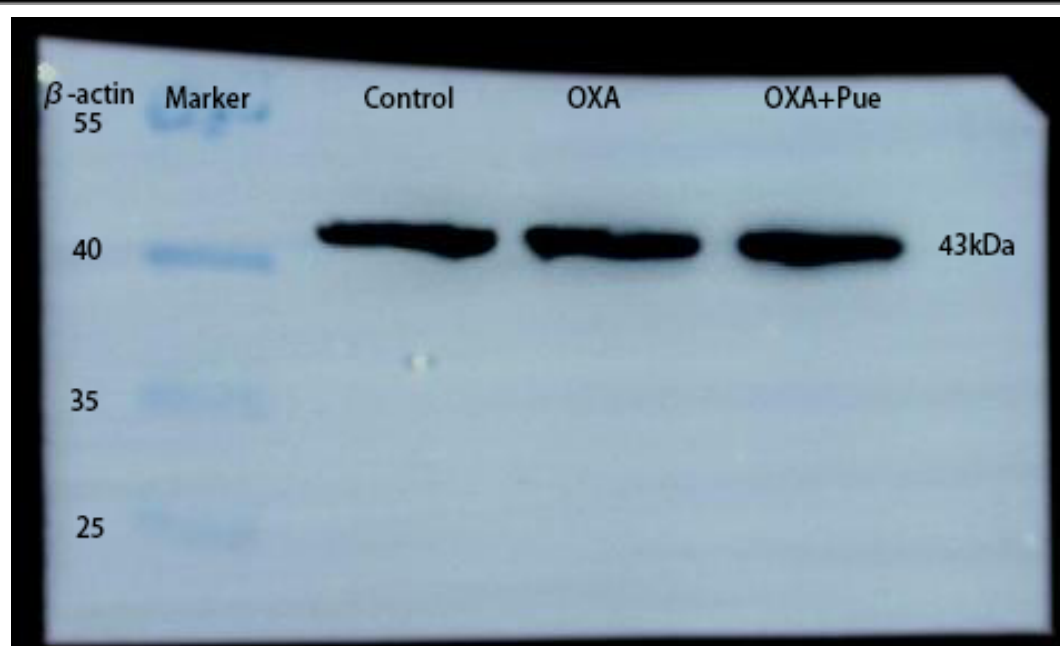

Fig 3

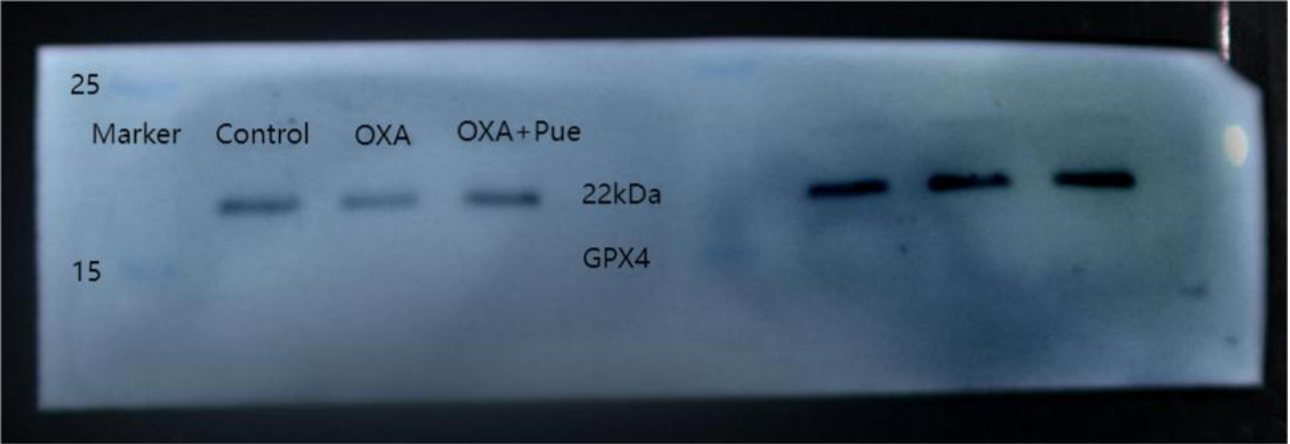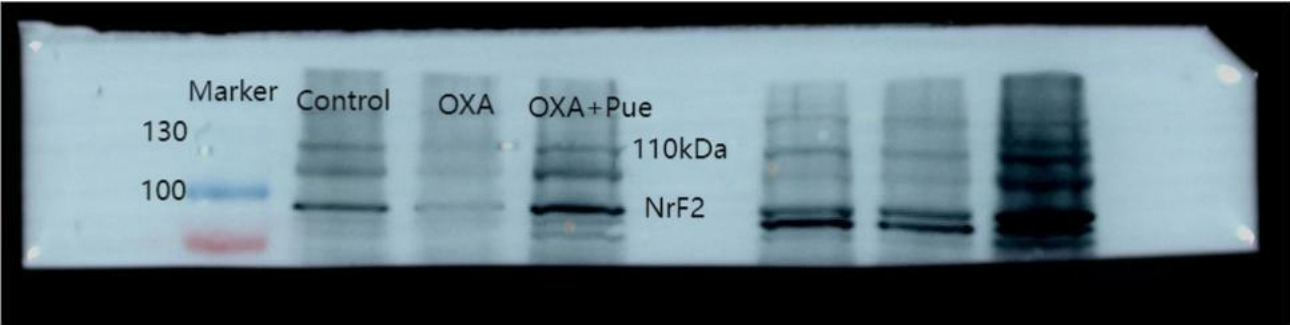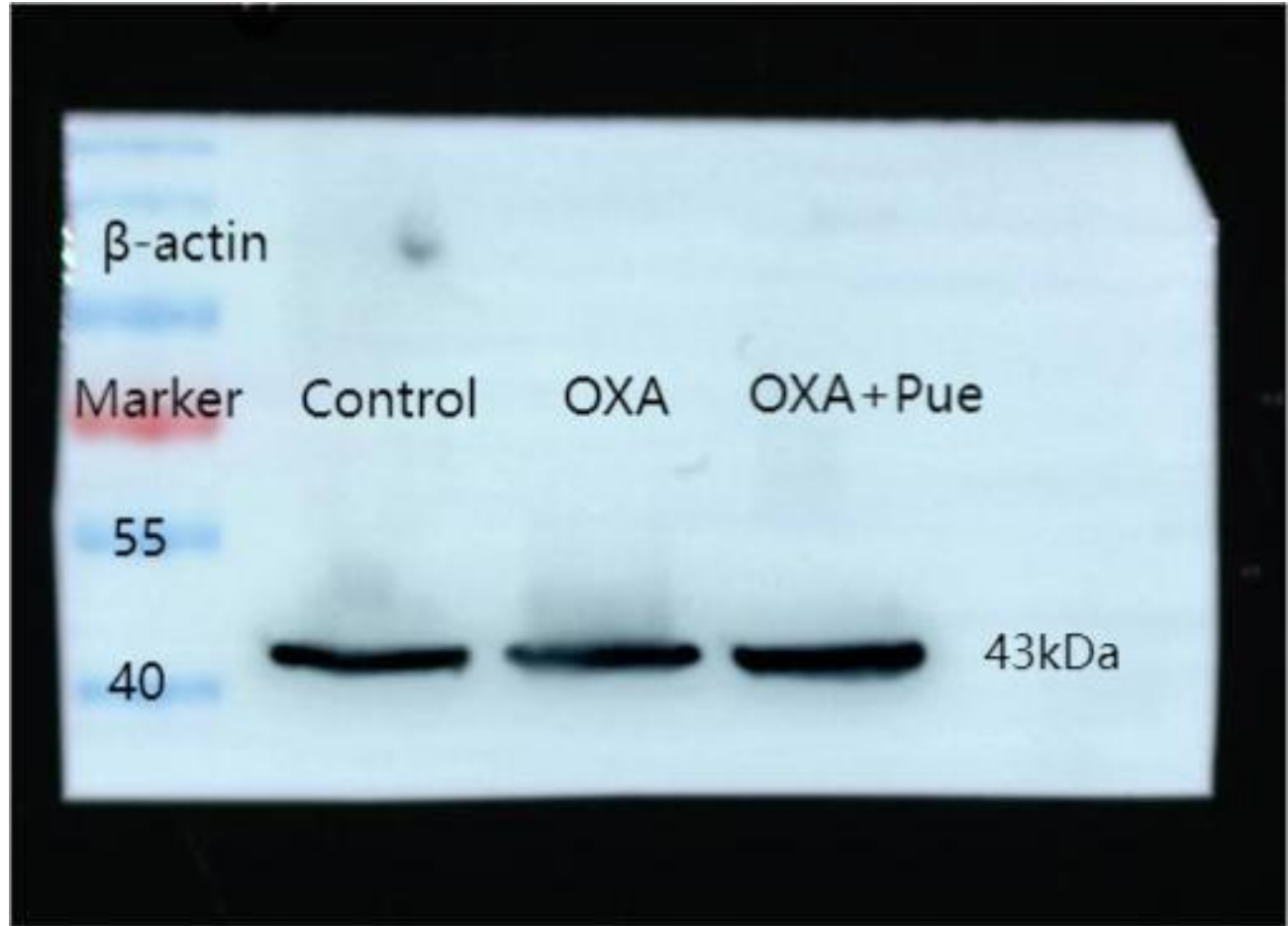

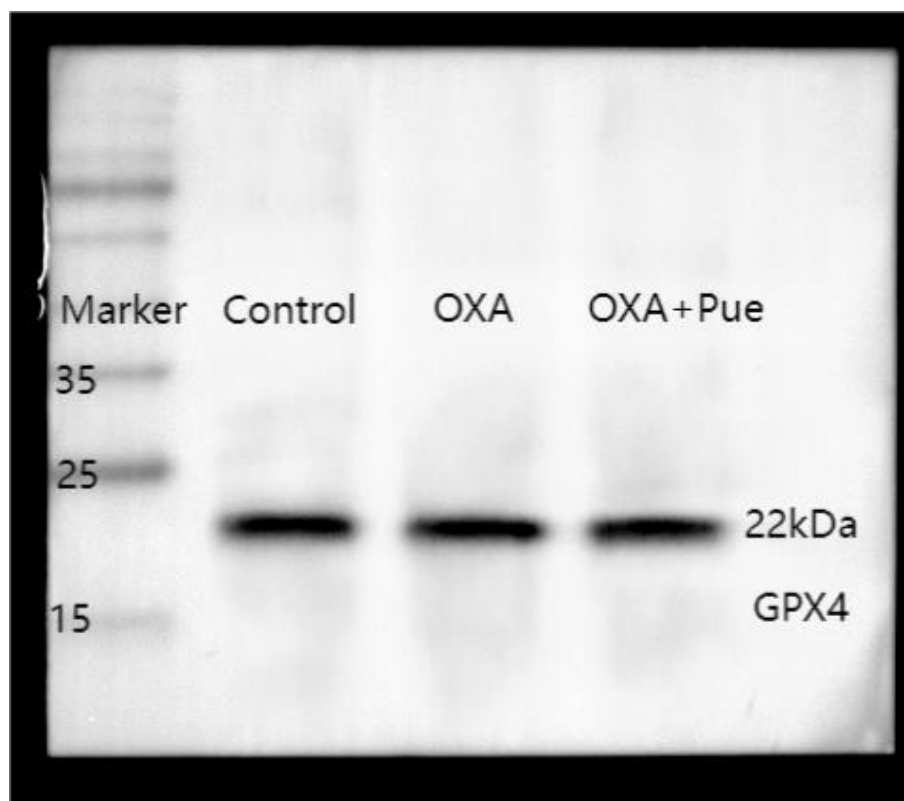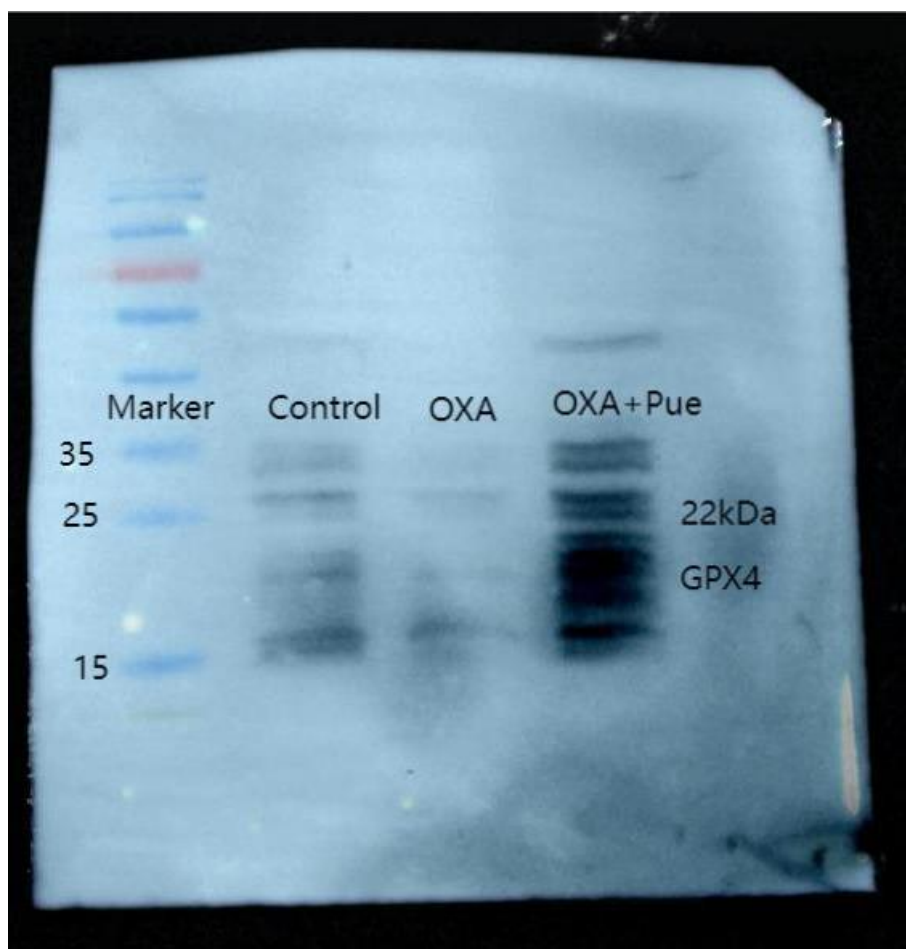

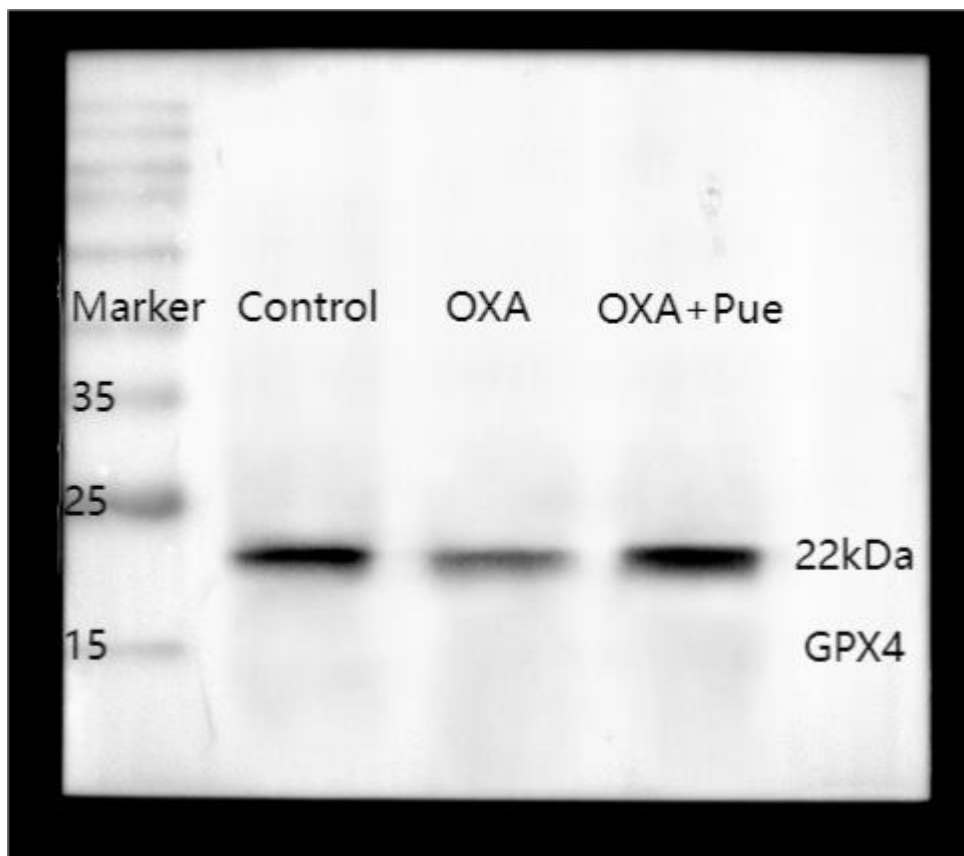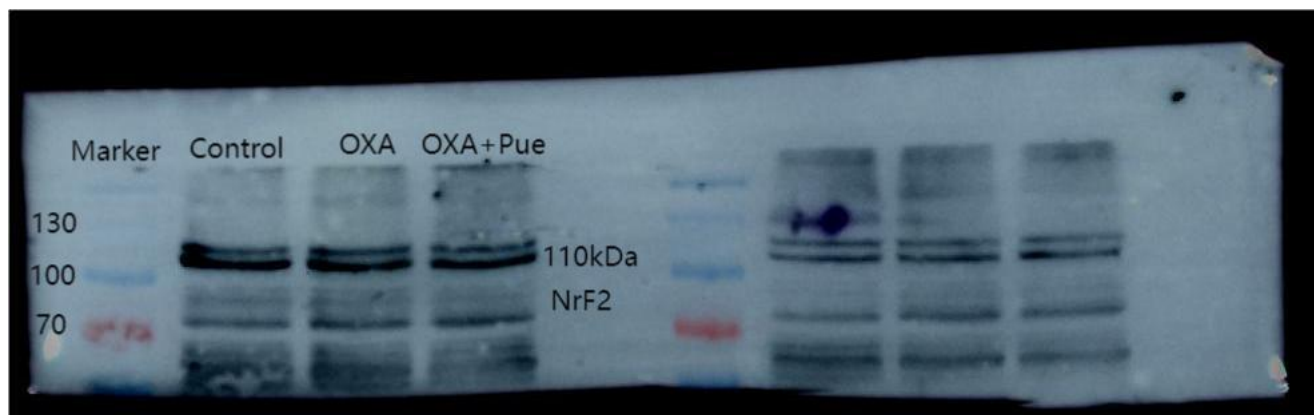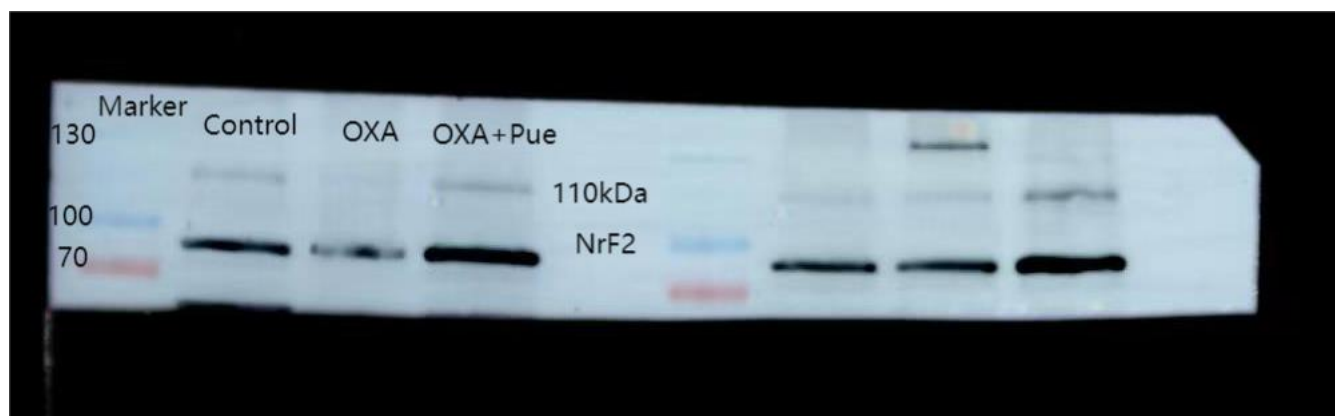

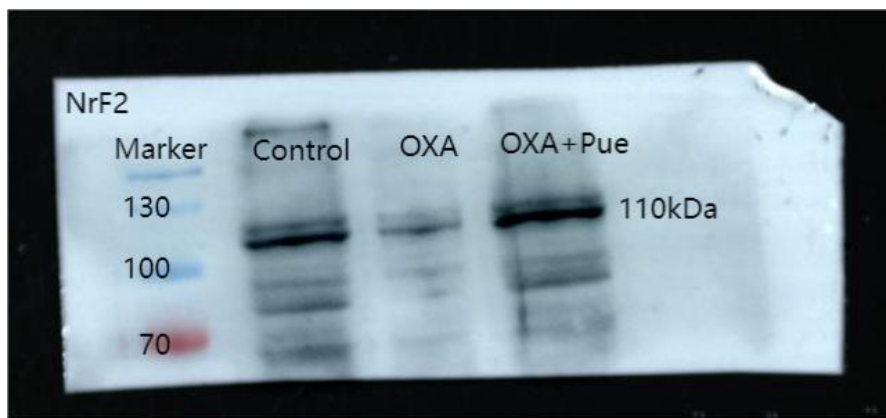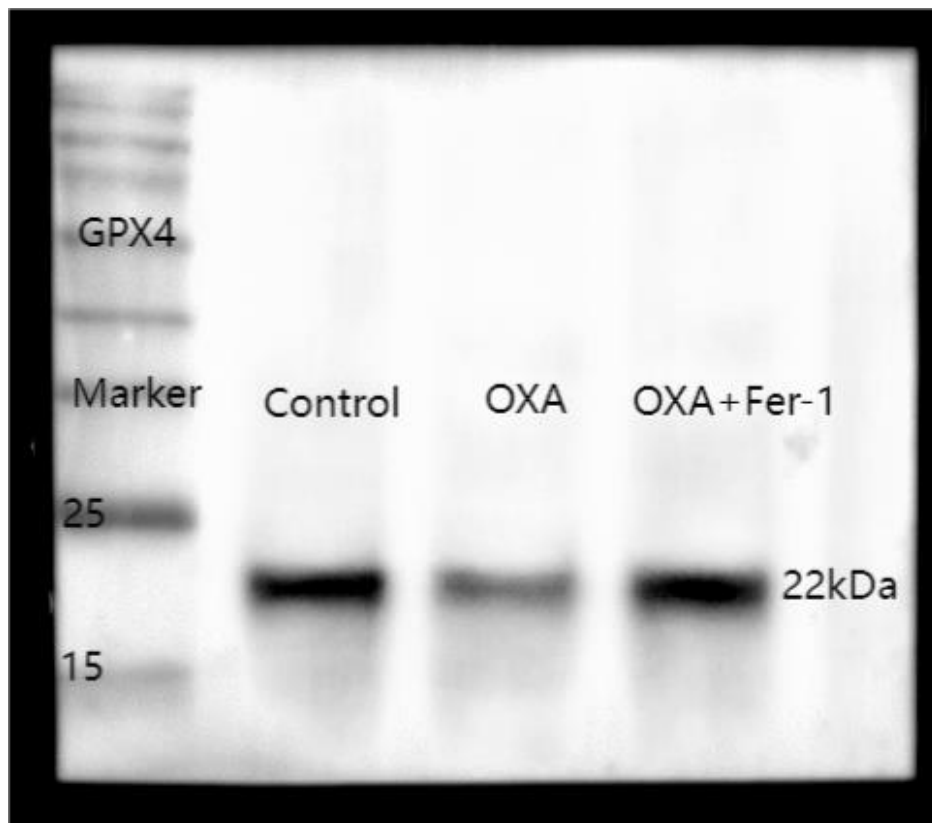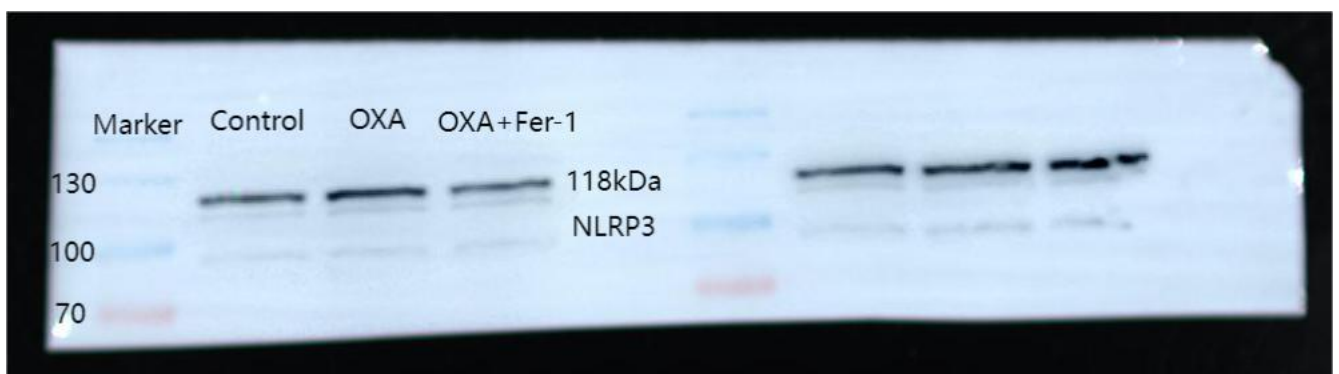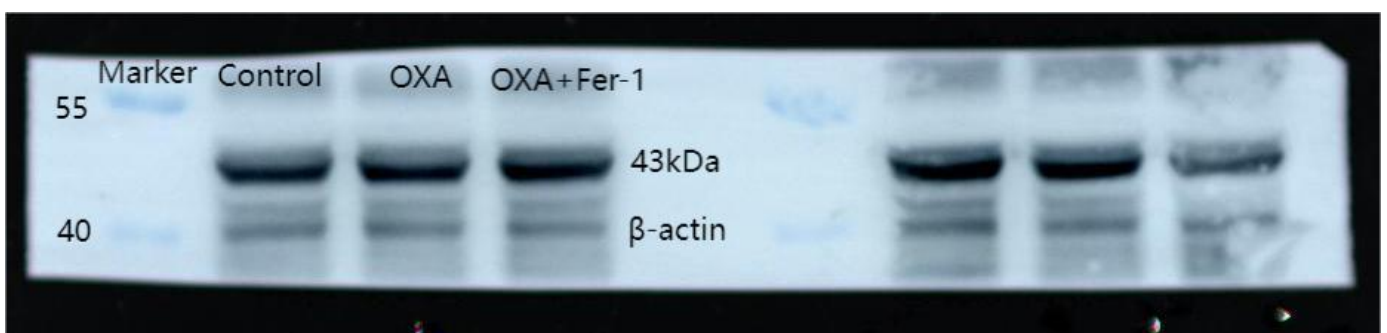

Fig 4

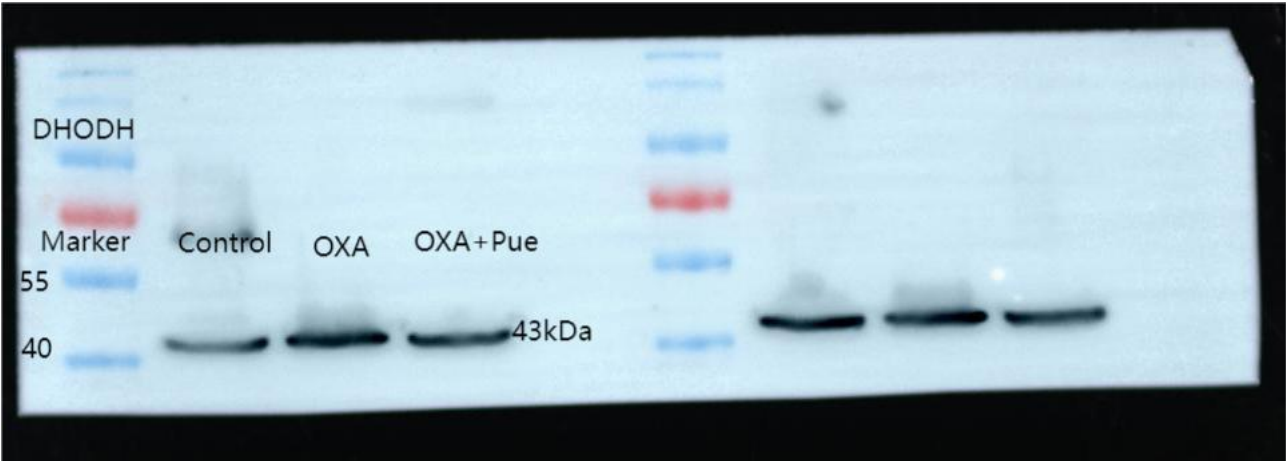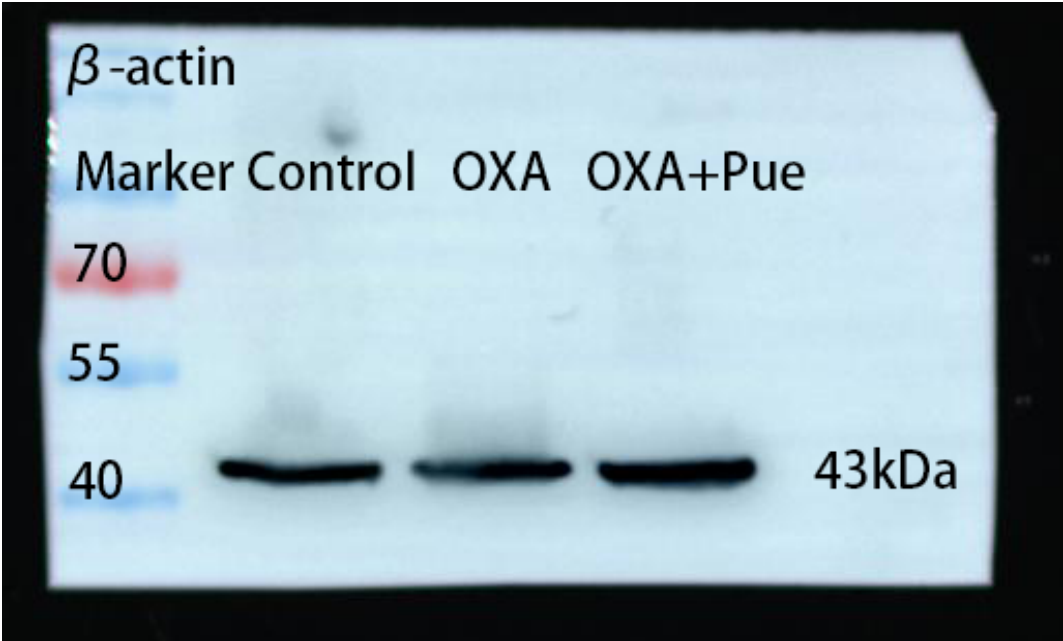

Supplement: S1 Raw image — (PDF) [file pone.0308872.s002.pdf]
